# 

**Published:** 1912-12-14

**Authors:** 


Leeds.—Tenders are invited for a twenty-bed sanatorium at Beckett Street. Messrs. J. Harper Bakes and Son are the architects. Tenders are also invited for additions and alterations at the workhouse adjoining from details prepared by Mr. G. F. Bowman, architect.

### Buildings Contemplated and in Progress.

Cambridgeshire.—It is proposed to extend the County Asylum buildings at Fulbourn, at a cost of £20,000, owing to complaints raised by the Lunacy Commissioners as to the inadequateness of the accommodation.

Devonport.—For sanction to a loan of £4,500 for purchase of land to extend the site of the infectious diseases hospital the Local Government Board has held an inquiry.

Dollis Hill.—An anonymous donor has entrusted Cardinal Bourne with £50,000 for the erection of a new Roman Catholic hospital at Dollis Hill.

Gloucester.—The Local Government Board has approved plans of a new infirmary submitted by the Guardians.

Goole.—A tender of £4,115 16s. 10d. has been accepted by the Guardians for alterations and additions to the workhouse premises.

Herts.—It is proposed to extend the County Mental Hospital at Hill End, and land therefor has been purchased at a cost of £4,273 16s.

Hornsey.—Tender forms may be obtained from the Borough Engineer's office for a laundry and disinfecting block at the Muswell Hill Isolation Hospital.

Johannesburg.—It is reported that the Union Government has given a site for a new institute for medical research. The Witwatersrand Native Labour Association is contributing £40,000 towards the cost of buildings and equipment.

Meltham.—The Urban District Council propose to extend the hospital at an estimated cost of £1,500.

Warminster.—Application is to be made to the Local Government Board for sanction to a loan of £3,600 by the Warminster Joint Hospital Committee.

### Gifts and Bequests.

The Trustees of the late Mr. Davies, of Glasgow, have promised £10,000 to Glasgow Victoria Infirmary out of the large sum which he left for charities.

Mrs. Aitkin, of Bodelwyddan Castle, has forwarded to the chairman of the committee of management of the Denbighshire Infirmary a cheque for £250 to cover the cost of an *x*-rays installation at the infirmary.

Among the latest contributions to King Edward's Hospital Fund for London are the following:—Annual subscriptions: Sir Savile Crossley, £250 (to capital); Messrs. Balfour, Williamson and Co., £105; Mr. George Lawson Johnston, £52 10s.; Mr. Frederick M. Fry, £25. Donation: Mrs. Sawbridge, £100.

The head office of one of the principal London banks states that a client has offered £5,000 to the Royal Free Hospital provided that an additional sum of £15,000 is subscribed or promised towards the building fund of the new extension before March 4, 1913. An appeal was recently issued for £50,000 needed to build and equip the new out-patient department. A tender for the construction of this building was accepted at the last meeting of the committee of management, and it is proposed that the work shall begin at once.

Mrs. Jane Ash, of Blackpool, has bequeathed the residue of her property, subject to various legacies, among the following institutions:—The Victoria Hospital, Blackpool, the Blackpool Sanatorium, the Devonshire Hospital and Buxton Bath Charity, the Manchester Royal Infirmary, Dispensary, and Lunatic Hospital or Asylum, the Northern Counties Supplementary Hospital for Chronic and Incurable Diseases, the Manchester Hospital for Consumption and Diseases of the Throat and Chest, Christie Hospital, Manchester, the Manchester Children's Hospital, St. Mary's Hospital, Whitworth Street, W., and Clifford Street, Manchester, and two other institutions. Mrs. Ash requested that each of the hospitals benefiting shall name a bed the "Mary Ann and Jane Higgin" bed, but she created no trust in that respect. The value of her charitable bequests will exceed £40,000.

### Appointments.

The Rev. Cecil J. Mead Allen has been appointed chaplain of Claybury Mental Hospital.

Dr. O. A. J. N. Muriset has been appointed chief tuberculosis officer for Northampton at a salary of £500 per annum.

Mr. E. S. Littlejohn, second assistant medical officer at Cane Hill Mental Hospital, has been promoted to be first assistant medical officer.

Dr. H. Neville Crowe, son of Dr. George W. Crowe, sen., honorary physician, has been elected to the honorary medical staff of Worcester General Infirmary, to fill the vacancy occurring on the resignation of Dr. Mabyn Read.

Dr. Harold Kerr, who has held the post of deputy medical officer of Newcastle, has been appointed medical officer, thus filling the vacancy occasioned by the resignation of Dr. Armstrong, at a salary of £800, increasing to £1,000.

Dr. Ivy E. Haslam, M.D., B.S., M.R.C.P. (Lond.), has been appointed honorary pathologist at the Warneford General Hospital, Leamington, and Mr. Frederick Sydenham, M.D., D.P.H., F.R.C.S. (Edin.), has been appointed honorary surgeon for diseases of the ear, nose, and throat. Mr. Wm. T. Dobson, M.R.C.S., L.R.C.P., has been appointed house physician.

Mr. T. Wingate Todd, M.B., Ch.B., F.R.C.S., Lecturer in Anatomy in the Manchester University, has been appointed Henry Willson Payne Professor of Anatomy in the Medical Department of the Western Reserve University, Cleveland, Ohio. Mr. Todd, who graduated in 1907 with first-class honours at the final examination for the degrees of M.B. and Ch.B., obtained amongst other distinctions whilst a student at the University the Tom Jones Scholarships in Anatomy and in Surgery. After graduation Mr. Todd was appointed Demonstrator in Anatomy under the late Professor Alfred H. Young. In 1909 he was elected house surgeon at the Manchester Royal Infirmary to Mr. Southam, who then occupied the Chair of Clinical Surgery in the University, and to Mr. J. W. Smith, the present holder of the Chair in Systematic Surgery.

### Books Received.

Daily News and Leader: "Year Book, 1913."

T. C. and E. C. Jack: "The Baby," by A University Woman.

Oxford Medical Publications: "Manual of Medicine," by A. S. Woodwark, M.D.

Baillière, Tindall and Cox: "Care of European Children in the Tropics," by Montagu Harston.

J. Wright and Sons: "A Manual of Infectious Diseases Occurring in Schools," by Armstrong and Fortescue Brickdale.

